# A single‐center study of thoracoscopic surgery in the treatment of pediatric mediastinal neurogenic tumors

**DOI:** 10.1111/1759-7714.14708

**Published:** 2022-11-09

**Authors:** Qiangye Zhang, Tingting Zhou, Peimin Hou, Weijing Mu, Dongming Wang, Jun Fang, Aiwu Li

**Affiliations:** ^1^ Qilu Hospital, Cheeloo College of Medicine Shandong University Jinan China; ^2^ Thoracic Surgery Department Children's Hospital Capital Institute of Pediatrics Beijing China; ^3^ Pediatric Orthopaedics Yidu Central Hospital of Weifang Weifang China

**Keywords:** mediastinal neurogenic tumors, children neurogenic tumor, thoracoscope

## Abstract

**Objective:**

To study the feasibility, safety, and efficacy of thoracoscopic surgery in the treatment of pediatric mediastinal neurogenic tumors, and summarize the treatment experiences and surgical skills.

**Methods:**

A single‐center retrospective analysis of 37 patients with pediatric mediastinal neurogenic tumors was conducted. Clinical charactersistics and postoperative complications were all analyzed.

**Results:**

All the operations were successfully completed. There was no statistically significant difference in tumor diameter between the two groups (*p* > 0.05). The open surgery group had an average operation time of 96.5 ± 32.38 min, while the thoracoscopic surgery group had an average operation time of 78.3 ± 24.51 min (*p* < 0.05). The thoracoscopic surgery group had significantly lower intraoperative blood loss than the open surgery group (*p* < 0.05). In addition, the duration of the postoperative thoracic drainage tube was 5.43 ± 0.76 days in the open surgery group, which was longer than the 2.38 ± 0.87 days in the thoracoscopic surgery group (*p* < 0.05). Furthermore, the postoperative length of hospital stay was an average of 10.23 ± 1.43 days for the open surgery group, longer than for the thoracoscopic surgery group (4.36 ± 0.87 days) (*p* < 0.05).

**Conclusions:**

Thoracoscopic surgery has several advantages in the treatment of pediatric mediastinal neurogenic tumors and is worthy of clinical popularization and application. For giant mediastinal malignant neurogenic tumors, puncture biopsy and adjuvant chemotherapy can be performed before surgery to lessen the tumor volume and enlarge the operation space, which would reduce bleeding and complications.

## Graphical abstract

Thoracoscopic surgery for the treatment of pediatric mediastinal neuroblastomas has numerous advantages, such as less trauma, faster recovery, less pain, and fewer postoperative complications, and deserves to be widely popularized and applied in clinical treatments.

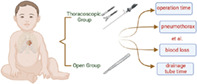

## INTRODUCTION

Neurogenic tumor is the most common primary posterior mediastinal tumor in children. It originates from primitive neuroectodermal neural crest cells and can occur in the adrenal gland, posterior mediastinum, neck, and pelvic cavity. Up to 15% of neurogenic tumors occur in the mediastinum.^1^ According to the differentiation and maturity of tumors, they are divided into neuroblastoma (NB), ganglioneuroblastoma (GNB), and ganglioneuroma (GN). Mediastinal neurogenic tumors have no typical clinical symptoms in the early stage.^2^, ^3^ NB is highly malignant, prone to early metastasis and poor prognosis,^4^ therefore early surgery is of great significance for the treatment and prognosis of pediatric patients. With the progress of minimally invasive thoracoscopic surgery, thoracoscopic resection has become the first‐line treatment for pediatric mediastinal tumors.

This study retrospectively analyzed the process of surgical treatments for 37 pediatric patients with neurogenic tumors which were verified by pathologists in Qilu hospital. Thus, we summarized the experience of single‐center treatments for pediatric mediastinal neurogenic tumors, and discussed the feasibility, efficacy, and safety of thoracoscopic surgery in this process.

## MATERIALS AND METHODS

### General information

We selectively performed a retrospective analysis of 37 children who underwent surgery at our pediatric surgery department from December 2015 to December 2021. All the guardians of the children signed the informed consent and acknowledged that the surgical data can be used for medical research purposes only. The principle for the seletion was based on final pathological diagnosis. General information on patients, including tumor size (diameter), preoperative imageology examinations, operation time, blood loss, postoperative tube‐carrying time, and postoperative length of hospital stay (from day of surgery to day of discharge), were all recorded and analyzed.

Open surgery was applied to resect mediastinal neurogenic tumors for 11 patients, including one case which was converted to open surgery (the surgical information for this case was recorded in the open surgery group), while thoracoscopic surgery was applied in the other patients. There were 11 children with an average age of 6.24 ± 1.4 years, comprising six males and five females, in the open surgery group and 26 children with an average age of 5.93 ± 1.7 years, comprising 14 males and 12 females, in the thoracoscopic surgery group. There were three cases with NB (27%) and eight cases with GN (73%) in the former group, and seven cases with NB (27%) and 19 cases with GN (73%) in the latter group.

### Surgical methods

#### Thoracoscopic surgery

**FIGURE 1:**
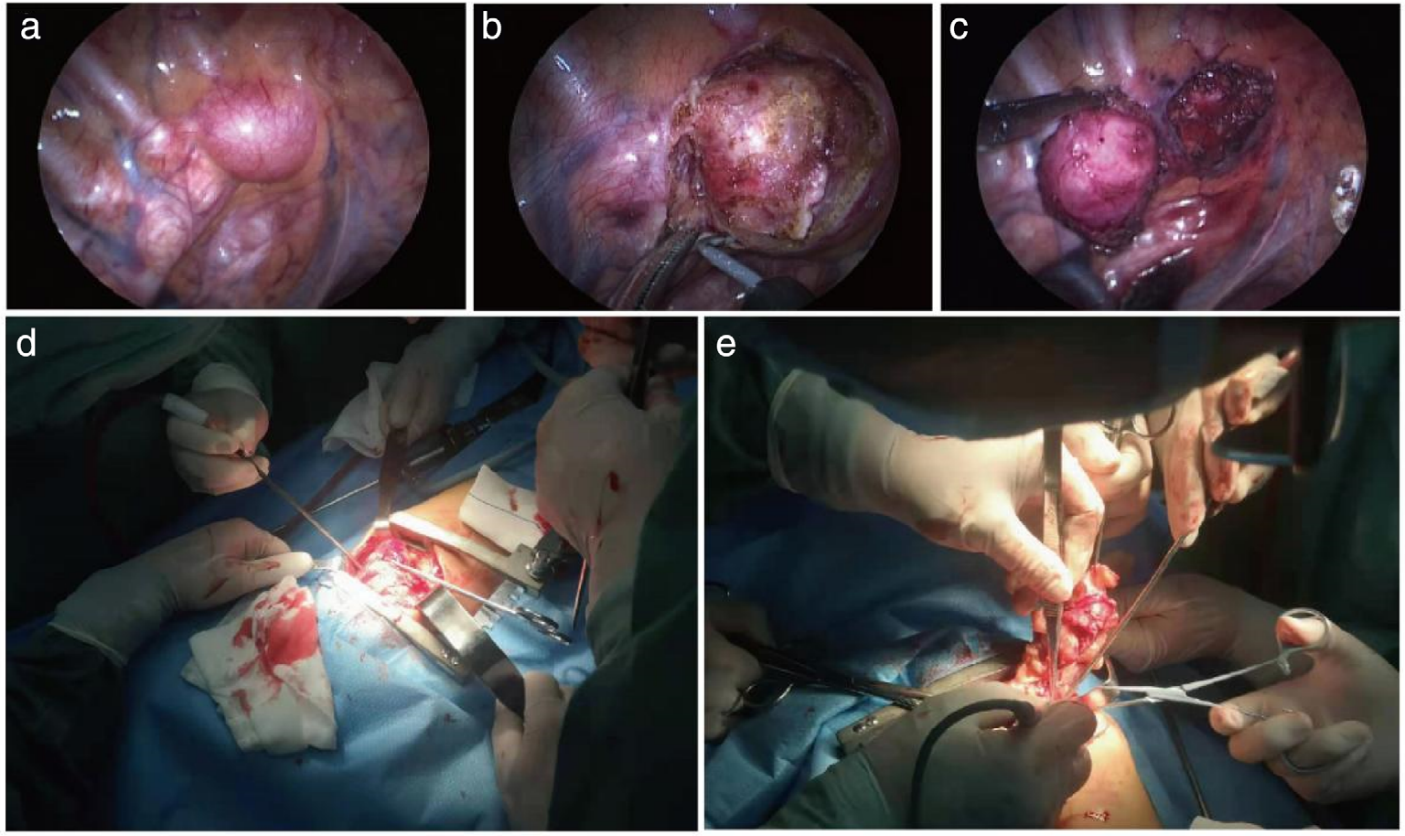
(a) Intraoperative exploration of a tumor located in the upper mediastinum. (b) Incision mediastinal pleura, free tumor. (c) Exact hemostasis, complete resection. (d) Open surgery for giant mediastinal tumor cannot be resected by thoracoscopic. (e) Resection of mediastinal tumor in blocks

After successful unilateral lung ventilation with endotracheal intubation under general anesthesia, pediatric patients were placed in the lateral decubitus position. The best position for patients during the operation was determained by the location of tumors to facilitate tumor exposure. In general, the lateral elevation position was the best choice for tumors located in the anterior mediastinum, while the lateral prone position was best for tumors located in the posterior mediastinum (Figure 1).

First, a 5‐mm trocar was placed at the intersection point between the fifth rib and the middle axillary line, then the thoracoscope was inserted through the trocar. The artificial pneumothorax pressure was set at 8‐10 mmHg. Before proceeding further the thoracic cavity was examined. Next, two 5‐mm trocars were placed at two intersection points between the fourth and sixth ribs, and the posterior axillary line. The specific positions for these two trocars were adjusted according to the tumor location and the detection. Detection of the area around chest and mediastinum was then performed to determine the relationship between the tumor and the heart, esophagus, trachea, and large blood vessels, as well as its boundaries and the conditions of adhesion. The mediastinal pleura was opened up (Figure 1a,b), the sidewall of the tumor was carefully isolated from the adjacent normal tissues (Figure 1c), and the exact hemostasis was required in each step. All these operations were performed on the premise of a clear vision. Finally, the tumor was completely removed through the trocar incision or an expanded one.

#### Open surgery

The procedure for the preparation for open surgery was similar to that for thoracoscopic surgery. The position for the thoracotomy was again determined by the location of the tumor, and exploration for the thoracotomy was conducted at the intersection point between the rib and the middle axillary line (Figure 1d). If the tumor was too large to be fully showed up, intercostal thoracotomy could be adapted to completely remove the tumor (Figure 1e).

### Preoperative imaging examination

Computed tomography (CT) is an important imaging method for the diagnosis of pediatric mediastinal neurogenic tumors that can accurately the nature of the tumor, its relationship with surrounding tissues and organs, and especially its invasion into blood vessels.^5^ All the children in the study with mediastinal masses underwent preoperative enhanced CT examination to evaluate the mass type and the relationship between the tumor and the adjacent vessels or organs.

**FIGURE 2:**
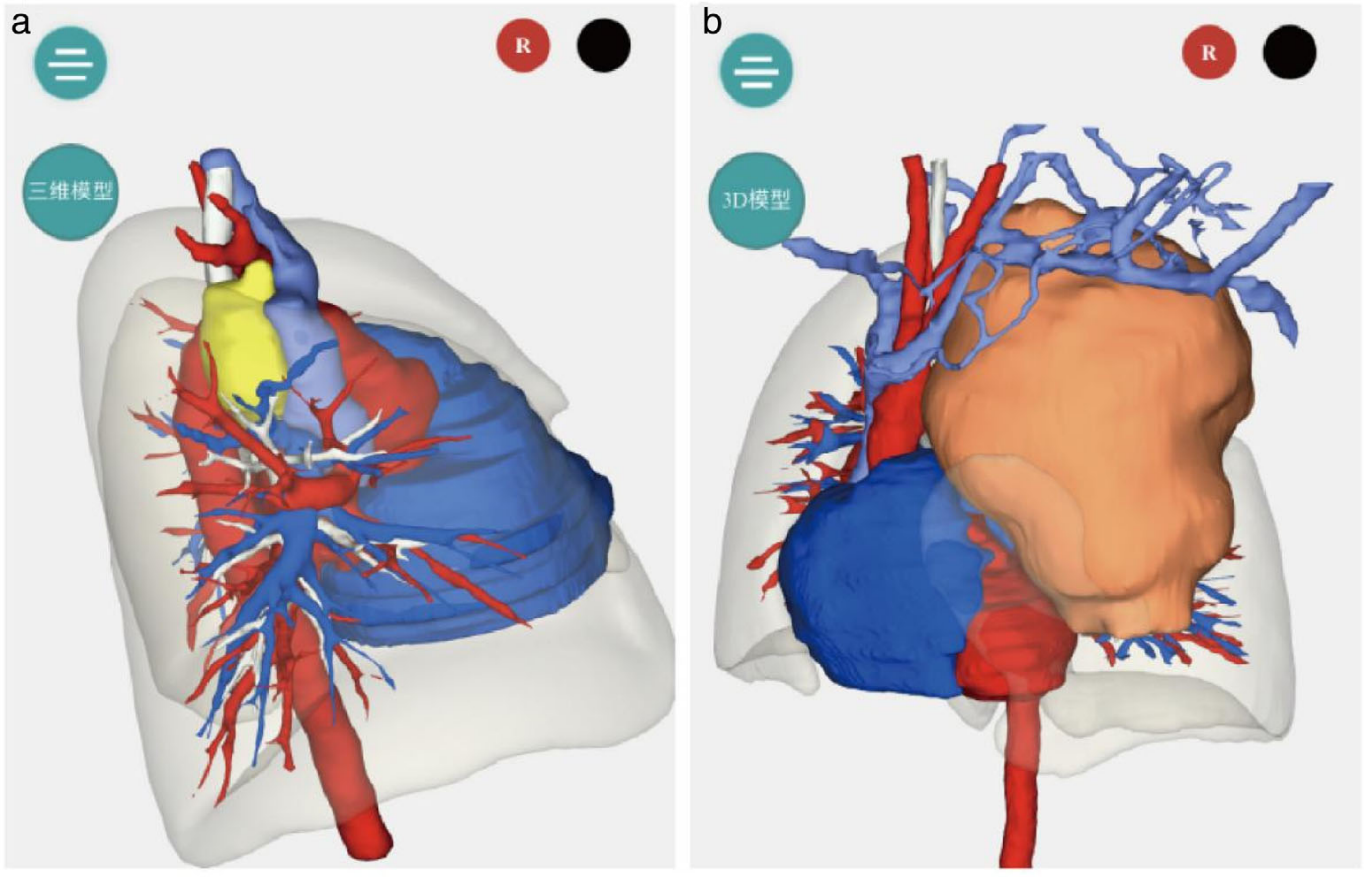
(a) Yellow portion of the mediastinal neurogenic mass clearly seen adjacent to the surrounding tissue. (b) Three‐dimensional reconstruction of neuroblastoma in one case of open surgery in this study shows large tumors

Evaluation of mediastinal tumors by three‐dimensional imaging reconstruction and printing technology has been reported.^6^ Establishing a preoperative three‐dimensional model helps surgeons to understand the blood supply of the tumor and its adjacent relationship, predict the underlying surgical risks, and determain the operation program.^7^ We utilized this advanced technology in this research to determine the size, location, and boundaries of the tumor, to gain a preliminary understanding of its relationship with the surrounding blood vessels, trachea, esophagus, and other organs as well as the adhesion condition of the surrounding tissues, and to provide an intuitive imaging basis for the evaluation of the patient's surgery (Figure 2).

Enhanced CT examination and three‐dimensional imaging technology have an important guiding significance in the diagnosis, tumor stage, and treatment options in mediastinal neuroblastomas.

### The application of ERAS

Enhanced recovery after surgery (ERAS) is applied in the perioperative period of thoracic surgery to reduce surgical complications. Warming the upper half of the body through the use of warming blanket during the operation reduces body surface exposure in children. Through air convection and radiation, the warm air is transmitted to the blanket via the pipeline by the air‐filled thermal insulation blanket, so that the heat is uniformly distributed on the surface covered by the blanket. It is necessary to block the intercostal nerves at the same site with the incision location and the adjacent upper and lower intercostal nerves together. This significantly reduces postoperative pain, increasing perioperative comfort and promoting patient rehabilitation.

### Statistical analysis

The statistical analysis was conducted using the SPSS 17.0 software. Counting data such as postoperative pneumo‐thorax, postoperative atelectasis or postoperative lung infection, etc. were expressed as percentages and tested by ANOVA analysis, while measurement data such as operation time, blood loss, postoperative tube carrying time, etc. were performed as mean ± SD, and statistically analyzed by *t*‐test.

## RESULTS

The operations were all successfully completed and all patients recovered well without any perioperative complications or death. The tumor in one patient was too large to be successfully removed by thoracoscopic surgery, so this operation continued by converting to open surgery, with blood transfusion required, thus this case was moved to the open surgery group.

The preoperative information for the two different surgery groups is shown in Table 1. Preoperative imaging showed no statistical significance in the gender, age and tumor diameters between the two groups (*p* > 0.05). The comparison of the two groups in Table 2 shows that thoracoscopic surgery has several advantages, such as less blood loss, faster recovery, and shorter hospital stay (*p* < 0.05).

**TABLE 1:** Comparison of data between the open surgery and thoracoscopic surgery groups

Surgery method	Gender		Age (years)	Tumor diameter (cm)	Tumer type	
	Male (*n* [%])	Female (*n* [%])			Neuroblastoma (*n* [%])	Ganglioneuroma (*n* [%])
Open surgery (*n* = 11)	6 (55%)	5 (45%)	6.24 ± 1.4	6.21 ± 1.27	3 (27%)	8 (73%)
Thoracoscopic surgery (*n* = 26)	14 (54%)	12 (46%)	5.93 ± 1.7	5.83 ± 1.41	7(27%)	19 (73%)
*p* value	*p* > 0.05					

**TABLE 2:** Comparison of surgical conditions and perioperative complications

Surgery method	Operation time (min)	Intraoperative blood loss (ml)	Postoperative thoracic drainage tube time (d)	Postoperative hospital stay (d)	Postoperative pneumo‐ thorax (*n* [%])	Postoperative atelectasis (*n* [%])	Postoperative lung infection [*n* (%)]	Postoperative self‐reported pain time (d)	Intraoperative blood transfusion (*n*)
Open surgery (*n* = 11)	96.5 ± 32.38	46.5 ± 16.8	5.43 ± 0.76	10.23 ± 1.43	1(9.1%)	2 (18.2%)	4 (36.3%)	4–5	1
Thoracoscopic surgery (*n* = 26)	78.3 ± 24.51	23.4 ± 11.72	2.38 ± 0.87	4.36 ± 0.87	2(7.6%)	1 (3.8%)	3 (11.5%)	2–3	
*p* value	*p* < 0.05								

There were two cases with a small amount of pneumothorax during the perioperative period in the thoracoscopy group, which disappeared after conservative therapy, as shown by X‐ray review. There was one case with pneumothorax in the open surgery group, which also disappeared after adjusting the position of the drainage tube, re‐fixing it and adopting conservative therapy, as shown by X‐ray review. The main reason for the postoperative pneumothorax was different in the two groups. In the thoracoscopic surgery group, it was caused by residual gas in the thoracic cavity after surgery because of the use of inflatable therapy during the operation, whereas in the open surgery group it was caused by large tumor size, an enlarged surgery wound, and the existence of lung injury. Fortunately, in both cases the postoperative pneumothorax was safely absorbed by conservative therapy instead of reoperation.

Additionally, there were two cases in the open surgery group and one case in the thoracoscopic surgery group with postoperative atelectasis, and four cases in the former group and two cases in the latter group suffered postoperative pulmonary infection. All the complications were resolved by applying a series of approaches, such as anti‐infection, aerosol inhalation, expectoration, pulmonary physiotherapy, etc. By contrast, it was found that the frequency of postoperative pulmonary symptoms for pediatirc patients in the open surgery group was significantly higher than in the thoracoscopic surgery group, which was thought to be related to the enlarged wound area, extended operation time, postoperative pleural adhesions, and so forth. In addition, open surgery required incision at the intercostal space and the ribs were kept apart during the operation, which caused stronger and longer‐lasting postoperative pain, consistent with the duration of pain shown in Table 2. As a result, patients could not cough deeply, which is not helpful in the subsidence of pneumonia and the lung recruitment.

In summary, thoracoscopic surgery for the treatment of pediatric mediastinal neuroblastomas has numerous advantages, such as less trauma, faster recovery, less pain, and fewer postoperative complications, therefore it should be considered for wider use in clinical treatments.

## DISCUSSION

Traditional thoracotomy for pediatric mediastinal neurogenic tumors can lead to great trauma, intraoperative bleeding, long postoperative hospital stays, and long drainage tube use times, all of which have a negative influence on respiratory and circulatory functions and lead to pain for patients.^8^ Recently, minimally invasive endoscopic surgery has developed rapidly and been widely applied, and video‐assisted thoracoscopy has gradually become popular in pediatric surgery.^8^, ^9^ Reports on thoracoscopic resection for pediatric mediastinal tumors are scarce in China and there is little experience of this technique, but in recent years, with the development of comprehensive treatments, its therapeutic effect in pediatric mediastinal neurogenic tumors has significantly improved.^10^ However, in terms of thoracoscopic treatments for mediastinal neurogenic tumors, standardized treatments on this aspect need further exploration and summary. In this research, through conducting a retrospective analysis of the distinct effect of two surgical methods on 37 pediatric patients with neurogenic tumors verified by pathologists, it is proposed that thoracoscopic surgery for pediatric mediastinal neurogenic tumors is worthy of clinical application.

### Preoperative evaluation of surgery selection and treatments for pediatric patients

**FIGURE 3:**
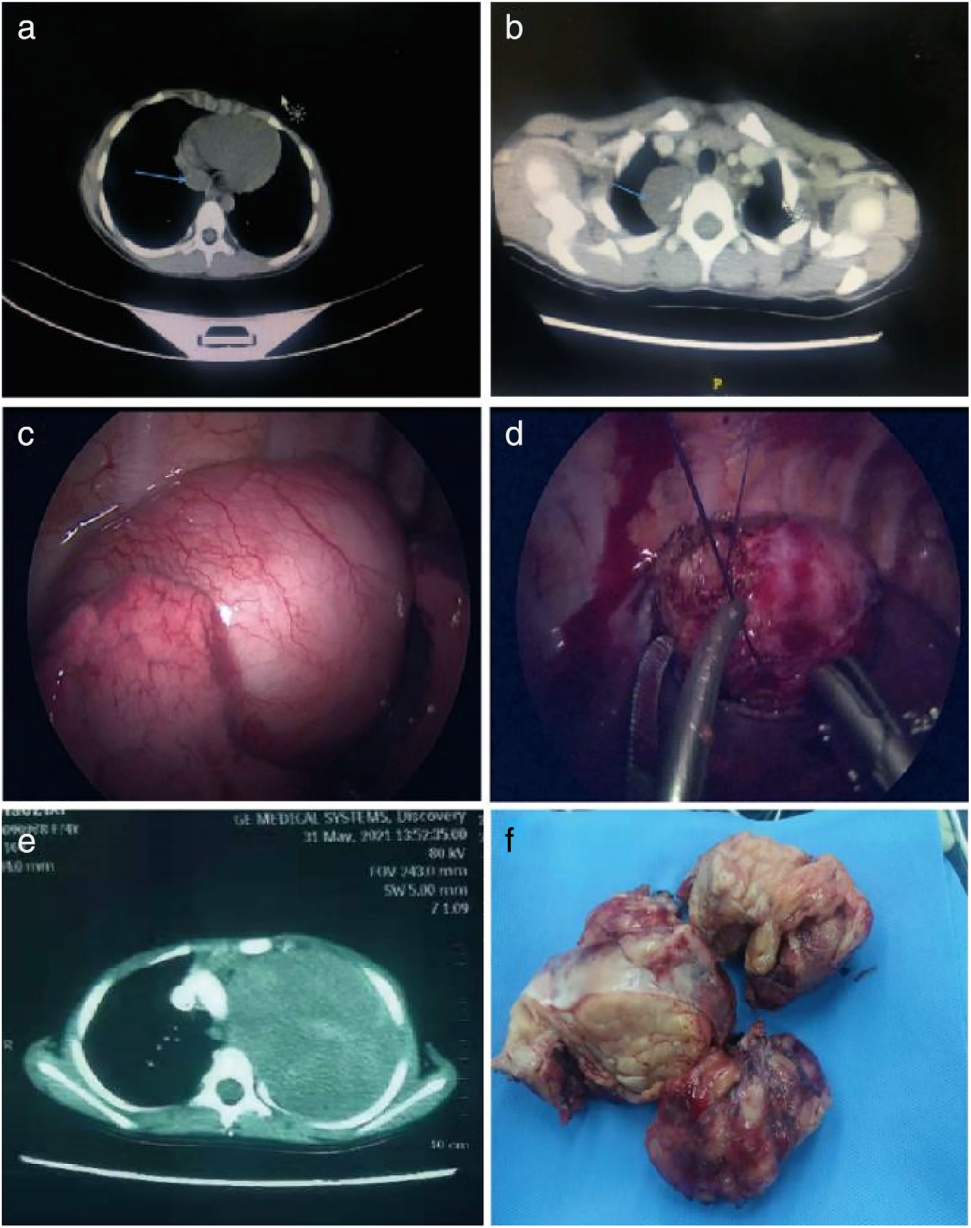
(a) Tumor/chest index ≤0.5. (b) Tumor/chest index ≥0.5. (c) The tumor is large and close to the spinal and macrovascular. (d) Suspended the tumor and exposed the gap. (e) Preoperative computerized tomography showing that the tumor is large and cannot be removed by endoscopy. Chemotherapy was started before surgery. (f) Piecemeal resection of the giant tumor by open surgery

There is no uniform standard for thoracoscopic indications. Previous studies suggested that endoscopic surgery was not suitable for children <6 months old, weight <8 kg, tumor/chest index (tumor diameter/tumor horizontal chest diameter) >0.5, tumor diameter >8cm, and tumors located at the costophrenic angle, top of chest, and neck–thorax junction.^11^, ^12^ However, with the gradual refinement of minimally invasive surgery and anesthesia, the scope for minimally invasive technology has increased. In this study, mediastinal masses located at the top of the chest and the junction of the neck and chest as well as mediastinal tumors with diameters up to 11 cm were successfully resected by thoracoscopic surgery. Therefore, according to preoperative imaging examinations, appropriate surgical methods can be selected on the basis of tumor size, especially neurogenic tumors, which are solid neoplasms with firm tissue and clear boundaries in the mediastinum. Selecting the most appropriate patient candidates can improve the chance of thoracoscopic surgery success, and reduce the proportion of conversion to thoracotomy and unnecessary trauma.^13^, ^14^ Our findings regarding patient selection are the following:

1. Thoracoscopic surgery can be successfully completed for mediastinal neurogenic masses with tumor size less than 10 cm.
2. Children with tumor/chest index (tumor diameter/tumor horizontal chest diameter) ≤0.5 can be selected for surgery.^12^, ^13^, ^14^ For children with tumor/chest index ≥0.5, thoracoscopic surgery is not absolutely suitable. The specific tumor characteristics, such as location at the relatively superficial position of the mediastinum, towards one side in the chest or with no important organ coating on its surface, will determine whether or not thoracoscopic surgery can be attempted (Figure 3a,b).
3. Pediatric patients with a tumor close to the macrovascular that cannot be removed by endoscopy surgery should lie on their backs for CT examination. Importantly, because the tumor will be closer to the macrovascular due to its own gravity, it was better that patients lie on their side to keep the tumor away from the macrovascular as far as possible, which would facilitate the thoracoscopic resection (Figure 3c). There are alternative ways to enlarge the space between the tumor and the macrovascular. For instance, intrathoracic air can be injected into the chest at a certain pressure or the tumor can be suspended on the chest wall. The tumor resection can be completely performed by thoracoscopic operation (Figure 3d).
4. Surgical treatment is not the first option for children with huge tumors. First, pathological verification for the neuroblastoma tissue obtained by puncturation under Ultrasound or CT localization should be performed. Thereafter, chemotherapy, which is one of the main treatments for high‐risk neuroblastoma, can be used to shrink the tumor before surgery as because most neuroblastomas are relatively sensitive to it. Studies have shown that compared with surgery, preoperative neoadjuvant therapy can usually reduce tumor volume and thus significantly improve the intact resection rate.^9^ In this study, there was one case with a giant tumor in the open surgery group. After preoperative evaluation, we decided to perform B‐ultrasound‐guided multipoint biopsy first and confirmed neuroblastoma. The whole lesion was easily resected after four rounds of chemotherapy (Figure 3e,f).
5. Thoracotomy should not be considered as a failure of minimally invasive surgery. The purpose of surgery is to extend the life span and improve life quality because survival is the first priority. In any case, the surgery should be implemented under the most safe situation, especially during the perioperative period. Children with giant mediastinal neuroblastomas should be treated with open surgery combined with medical therapy, namely, preoperative chemotherapy–surgical resection–postoperative chemotherapy, which is known as the ‘sandwich’ method, and is the most appropriate treatment.

### Application of ERAS

Lately, the concept of ERAS has gradually emerged. This is a multidisciplinary model that adopts a variety of evidence‐based perioperative interventions, including optimizing surgical operation, anesthesia technology, postoperative analgesia, postoperative nutrition, and organ support, to accelerate the recovery of patients after surgery.^15^ Hence, we applied ERAS principles throughout the procedures to reduce trauma and aid patient recovery.

#### The importance of heat preservation during surgery

In pediatric thoracoscopic surgery, hypothermia is prone to occur, mainly due to the loss of the behavioral regulation mechanism in children during general anesthesia and the inhibition of the central regulation system because of temporary hypogenesis of body temperature by anesthetics.^16^ Improper intraoperative thermal insulation will lead to adverse events such as hypothermia and chills. Because of the incomplete development of their body temperature regulation center, children have poor ability to regulate body temperature and are greatly affected by the external environment. Since the upper body is the surgical site, the disinfection area needs to be expanded and therefore the exposure area is large in pediatric patients. In addition, the long operation time also increases the risk of hypothermia for children. By using a warming blanket during the operation and improving the surface temperature, heat diffusion from the body to the surrounding environment was reduced, thus increasing the heating efficiency, which plays a significant role in heating.

#### The painless experience of children^17^

At present, patient‐controlled intravenous analgesia (PCIA) is commonly used for postoperative analgesia after pediatric thoracic surgery. However, PCIA should be continuously pressed to increase the dosage of opioid analgesics during the peak period of inflammatory factor release within 24 h after operation. Therefore, anesthesiologists should choose an ultrasound‐guided intercostal nerve block analgesia during surgery.

#### Selection of surgical methods to reduce trauma and operation time

During the perioperative period, the standards for minimal invasiveness, safety, and radical tumor resection should follow the principles of safety first and prioritize radical tumor therapy.^18^ Because thoracoscopic resection of mediastinal mass is difficult, sufficient preparations must be made before surgery. If an abnormal situation is encountered during the operation, the operation should be accurately evaluated. If necessary, thoracotomy surgery should be decided in time, and it is not appropriate to complete the operation by the thoracoscope.

## CONCLUSION

When a mediastinal neurogenic tumor is found in a pediatric patient, the necessary examinations should be completed as soon as possible, including preoperative evaluation. Through retrospective analysis, we concluded that thoracoscopy in the treatment of pediatric mediastinal neurogenic tumors ha a clear vision to observe the relationship between the tumor and large blood vessels and/or surrounding organs, especially in the case of mediastinal masses at the top of the chest and the junction of the neck and chest. Thoracoscopic surgery has many advantages, such as less trauma, faster recovery, and better safety than traditional open surgery.

For malignant tumors that were previously considered to be unable to be dealt with by endoscopy, the relationship between the tumor and blood vessels can be clarified by preoperative three‐dimensional imaging. During the operation, the tumor can be pushed to one side due to its own gravity when the patient lies on his or her side, thus enlarging the space between the tumor and the thoracic aorta. In addition, the tumor can be suspended onto the chest wall during the operation, and complete thoracoscopic resection can be performed using this technology. For giant neuroblastoma, puncture biopsy and adjuvant chemotherapy should be performed first. When the tumor shrinks and the operating space in the chest becomes larger, thoracoscopic surgery can be conducted to reduce bleeding and complications. In summary, thoracoscopic surgery is worthy of wide clinical application in the treatment of pediatric mediastinal neurogenic tumors.

## CONFLICT OF INTEREST

The authors declare that the research was conducted in the absence of any commercial or financial relationships that could be construed as a potential conflict of interest.

## ETHICS STATEMENT

The study was approved by the Ethics Committee of Qilu Hospital, Shandong University. All procedures involving human subjects were conducted in accordance with the ethical standards of the institution.
